# Generating golden Syrian hamsters with conditional alleles via zygote microinjection of CRISPR/Cas9

**DOI:** 10.1002/ame2.70107

**Published:** 2025-11-14

**Authors:** Wei Chen, Xu Zhang, Rui Fan, Xia Li, Feifei Guan, Gefan Wan, Weining Kong, Xiaolong Qi, Shuo Pan, Sijing Shi, Yuanlong Su, Shan Gao, Wei Huang, Xunde Xian, Jiangning Liu, Yuhui Wang, Yuanwu Ma

**Affiliations:** ^1^ State Key Laboratory of Respiratory Health and Multimorbidity, NHC Key Laboratory of Human Disease Comparative Medicine, Key Laboratory of Pathogen Infection Prevention and Control, Ministry of Education, National Human Diseases Animal Model Resource Center and National Center of Technology Innovation for Animal Model Institute of Laboratory Animal Science, Chinese Academy of Medical Sciences (CAMS), Peking Union Medical College (PUMC) Beijing China; ^2^ Institute of Cardiovascular Sciences, State Key Laboratory of Vascular Homeostasis and Remodeling, School of Basic Medical Sciences Peking University Beijing China; ^3^ Department of Laboratory Animal Science Peking University Health Science Center Beijing China; ^4^ Haihe Laboratory of Cell Ecosystem Tianjin China; ^5^ Medical Primate Research Center, Chinese Academy of Medical Sciences Beijing China

**Keywords:** conditional knockout, genome editing, Golden Syrian hamster

## Abstract

**Background:**

The golden Syrian hamster is a valuable animal model for studying carcinogenesis, metabolic disorders, cardiovascular diseases, and viral infections due to its biological and pathological similarities to humans. However, the development of genetically engineered hamsters has lagged behind that of mice and rats, largely because of an embryonic development block at the two‐cell stage in vitro. Although CRISPR/Cas9‐mediated gene knockout has been achieved in hamsters, precise DNA fragment insertion or conditional knockout (cKO) models have not previously been reported, likely due to technical limitations in embryo manipulation and insufficient efficiency of homology‐directed repair (HDR).

**Methods:**

In this study, we generated conditional alleles of the *ApoF* gene in golden Syrian hamsters. A two‐cut strategy was applied using Cas9 protein, two sgRNAs, and a single donor plasmid containing exon 2 flanked by *loxP* sites and two ~0.8 kb homology arms. A mixture of Cas9 protein, sgRNAs, and the donor plasmid was microinjected into the pronuclei of one‐cell stage hamster embryos.

**Results:**

The efficiency of CRISPR/Cas9‐mediated *loxP* knock‐in reached up to 27%, and the genetically modified floxed alleles were successfully transmitted through the germline. The functionality of the inserted *loxP* sites was validated by in vivo Cre‐mediated recombination following local administration of AAV vectors, including AAV‐cTnT‐Cre in the heart and AAV‐CMV‐Cre in the brain.

**Conclusions:**

To our knowledge, this work represents the first successful establishment of a conditional knockout model in the golden Syrian hamster, providing a valuable tool for mechanistic studies of gene function and disease modeling.

## Graphical abstract

We established the first conditional knockout (cKO) model in the golden Syrian hamster by CRISPR/Cas9‐mediated genome editing. Cas9 protein, two sgRNAs, and a donor plasmid carrying loxP‐flanked exon 2 of the ApoF gene were microinjected into one‐cell embryos. The floxed allele was efficiently generated (up to 27%) and transmitted through the germline. Functionality of the loxP sites was confirmed by Cre recombination in vivo after AAV‐Cre delivery to the heart and brain. This work provides a valuable genetic tool for mechanistic studies and disease modeling in hamsters.

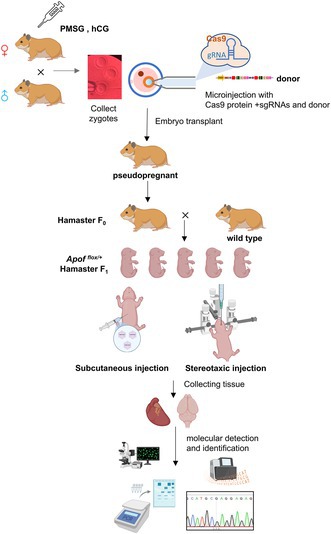

## 1 INTRODUCTION

The golden Syrian hamster (also known as golden hamster, *Mesocricetus auratus*) has long been recognized as a valuable model for biomedical research, particularly in the fields of reproduction,^1^ oncology,^2^ metabolic disorder,^3^ cardiovascular disease,^3^ and virus infections.^4^ Biological and pathological similarities to humans, including high levels of cholesteryl ester transport protein (CETP), low levels of hepatic low‐density lipoprotein (LDL) receptor activity, a high glycemic response to dietary fructose,^5^ and unique susceptibility to human viral pathogens (e.g., SARS‐CoV‐2),^4^ make the golden Syrian hamster a valuable model for studying human diseases. Despite these prominent strengths, the application of advanced genetic engineering techniques in hamsters has largely fallen behind in other rodent models, such as mice and rats.

A significant bottleneck hindering genetic manipulation in hamsters is the in vitro developmental block of embryos at the two‐cell stage.^5^, ^6^, ^7^, ^8^ This fundamental challenge has severely hampered the application of zygote microinjection‐based strategies that are routine in mice (*Mus musculus*)^9^, ^10^ and rat (*Rattus norvegicus*).^11^, ^12^ Although the advent of the CRISPR/Cas9 system has revolutionized genome editing and facilitated the generation of gene knockout hamsters by several research groups,^5^, ^13^, ^14^, ^15^, ^16^ more sophisticated genetic modifications remain largely unexplored. The improved genome editing via oviductal nucleic acids delivery (i‐GONAD) method enables direct in vivo genome editing within the oviduct. This approach overcomes the two‐cell block encountered in vitro, leading to a higher yield of genetically modified hamsters.^7^ However, the homology‐directed repair (HDR)‐mediated insertion of precise DNA fragments, most notably, the creation of conditional knockout (cKO) models—a cornerstone of modern genetic analysis for studying gene function in a spatiotemporal‐specific manner—has not yet been reported. The reasons for this gap are likely multifaceted, potentially involving suboptimal efficiency of HDR in hamster zygotes.

Currently, the genetic toolbox for hamsters is primarily limited to constitutive knockouts.^3^, ^7^, ^13^, ^14^ The absence of a reliable conditional allele system prevents tissue‐specific or inducible gene ablation, thereby limiting investigations into genes essential for development or those with pleiotropic functions. This critical technological gap restricts the full potential of the hamster model for sophisticated mechanistic studies. Here, we report for the first time successful establishment of a conditional knockout golden Syrian hamster. We designed a strategy utilizing Cas9 protein, dual sgRNAs, and a single donor template plasmid containing a *loxP*‐flanked critical exon flanked by homology arms. By microinjecting the editing mixture into fertilized hamster eggs, we achieved highly efficient generation of floxed alleles, with efficiency up to 27%. These conditional alleles were successfully transmitted through the germline and functionally validated by Cre‐mediated recombination in vivo via AAV‐Cre delivery into the heart and the brain. Our work breaks the technical barrier that has long impeded advanced genetic modeling in hamsters. This work provides the research community with a vital toolset to perform more precise genetic manipulations, thereby unlocking the hamster's full potential as a powerful model for deciphering human disease mechanisms.

## 2 METHODS

### 2.1 Animals

Wildtype golden Syrian hamsters were purchased from Vital River company (Vital River Laboratory Animal Technology Co., Ltd., Beijing, China) and housed in standard cages and maintained on a 14 h light and 10 h dark cycle (daily light period, 7:00–21:00), free access to food and water. All animal experiments were approved by the Animal Care and Use Committees (IACUC) of the Institute of Laboratory Animal Science, Peking Union Medical College (IACUC‐MYW23004).

### 2.2 DNA constructs

The sgRNA target sites were designed and prepared according to previously established protocols.^17^ Briefly, the sgRNA expression plasmids were prepared by inserting the oligonucleotides of designed sgRNA into the pUC57‐sgRNA plasmid. The sequences of oligonucleotides used are provided in Table 1. The ApoF‐floxed donor template plasmid containing a *loxP*‐flanked exon 2 flanked by two ~0.8 kb homology arms was designed and constructed based on the golden hamster genomic sequence (assembly BCM_Maur_2.0) and subsequently cloned into the pGSI vector.

**TABLE 1:** Oligonucleosides used for sgRNA preparation.

Primer	Sequence (5′‐3′)
GH‐ApoF‐E2A‐gRNA up	TAGGTTGGAGGTAGGTGATGGG
GH‐ApoF‐E2A‐gRNA dw	AAACCCCATCACCTACCTCCAA
GH‐ApoF‐E2B‐gRNA up	TAGGCAAGAGCAGGAGCATAGT
GH‐ApoF‐E2B‐gRNA dw	AAACACTATGCTCCTGCTCTTG

### 2.3 The sgRNA preparation

Following digestion with Dra I, the linearized sgRNA plasmid served as the template for in vitro transcription, which was carried out using the MEGAshortscript™ Kit (AM1354, Ambion, Thermo Fisher Scientific Inc., Waltham, MA, USA) and purified with the MEGAclear™ Kit (Ambion, AM1908).

### 2.4 Superovulation, microinjection, and embryo transfer in syrian golden hamsters

Six‐week‐old golden Syrian hamsters (Vital River Laboratory Animal Technology Co., Ltd., Beijing, China) were acclimated for one week before experiments. Adult golden Syrian hamsters were maintained under controlled environmental conditions with a 14‐h light and 10‐h dark cycle (lights on 07:00–21:00) and provided with food and water ad libitum. Females on the first day of estrus were injected intraperitoneally with 30 IU of PMSG at 10:00 a.m., followed by 30 IU of HCG on day 4 at 10:00 a.m., and then mated with males. On the same evening, additional estrous females were paired with vasectomized males under red light, and those confirmed to have mated were housed individually for subsequent use. Golden hamster embryos are highly sensitive to visible light exposure, and earlier studies demonstrated that such exposure can markedly reduce in vitro development.^6^, ^18^ Oocytes were collected from the oviductal ampullae 12–16 h after HCG administration (i.e., between 22:00 and 02:00) to ensure recovery of mature M2 oocytes. All procedures, from zygote collection to embryo transfer, were conducted under red light (>620 nm) to avoid detrimental effects of white light exposure. For zygote collection, mated females were euthanized and the oviducts were excised and dissected in M2 medium to release cumulus–zygotes complexes, which were treated with 2 μL hyaluronidase for approximately 5 minutes to remove cumulus cells. Denuded zygotes were washed three times in fresh M2 medium and transferred into HECM‐10 medium pre‐equilibrated overnight in an incubator at 37.5℃ under 10% CO_₂_ and 5% N_₂_. Microinjection was performed following the same protocol as for rats and mice.^19^ Prior to microinjection, the prepared samples were centrifuged at 14 000 rpm for 50 min and kept on ice. For injection, a 50 μL drop of M2 medium was placed on a culture dish lid and covered with mineral oil to form a droplet chamber, which was positioned on the stage of the micromanipulation system. A holding pipette was pulled using a needle puller, cut to the desired diameter with a microgrinder, closed with a microforge, and bent at an appropriate angle before being mounted on the injection arm and adjusted for position and angle. Approximately 50 fertilized eggs were transferred into the injection chamber and injected within 30 min. Following injection, the embryos were extracted into HECM10^20^ medium and returned to the incubator for further culture prior to transfer (microscope: Eclipse Ti, Nikon, Japan; micromanipulator: Narishige, Nikon, Japan; micropipette puller: PN‐30, Narishige, Japan).

The embryos were transplanted into the fimbria of the fallopian tube. The female mice selected the day before and mated with the castrated male mice were anesthetized and shaved on the back. A slit about 1 cm was made, and the fallopian tubes were exposed on both sides of the inner epidermis layer by opening the slit. The transplantation surgery was performed under a dissecting microscope, and then a few drops of antibiotics were added before suturing. The embryos were then placed on a warming table for incubation until they woke up.

### 2.5 Genomic DNA preparation

Genomic DNA purification from 7‐day‐old hamster tails was performed with the EasyPure® Genomic DNA Kit (EE101, TransGen Biotech, China). The purified DNA was then resuspended in TE buffer and stored at −20℃ until further use.

### 2.6 Germline transmission assay

The F_0_ founder (#10) was crossed with a wild‐type hamster to test the germline transmission of the engineered alleles. The F_1_ offspring's genomic DNA was extracted and genotyped using PCR and subsequent Sanger sequencing. A mutation detected in F_1_ progeny matching that of the F_0_ parent was considered as successful germline transmission. Primers used are listed in Table 2.

### 2.7 AAV production and packaging

Recombinant adeno‐associated viruses (AAVs) were commercially packaged and provided by Obio Technology Corp., Ltd., Shanghai, China. The following AAVs were used: AAV‐cTnT‐Cre (E10937): encoding Cre recombinase under the control of the cardiac‐specific troponin T (cTnT) promoter; AAV‐cTnT‐mCherry (H10844): encoding the mCherry fluorescent reporter driven by the cTnT promoter; AAV‐CMV‐Cre‐EGFP (CN396): encoding Cre recombinase and EGFP under the control of a CMV promoter. Viruses were packaged using AAV2/9 serotype, with final titers of [1 × 10^12^ v.g./mL]. Aliquots were stored at −80℃ in the dark and thawed on ice immediately prior to use.

### 2.8 In vivo AAV‐Cre mediated recombination in *ApoF cKO* hamster

To detect the activity of *Cre/loxP*‐mediated recombination in vivo, AAV packaged with Cre recombinase was administered. In vivo *Cre*/*loxP*‐mediated recombination was performed by local administration of AAV‐Cre in heart and brain.

For heart delivery, four‐day‐old hamsters were anesthetized using hypothermia, and 9 μL of AAV (1 × 10^12^ v.g./mL) was delivered into the subcutaneous space of the anterior chest using a SGE010RNS (10 μL syringe with replaceable 26 g × 50 mm beveled needle; Trajan Scientific and Medical, Melbourne, Australia) inserted at a shallow angle. Successful injection was confirmed by a small bleb under the skin, and pups were kept warm until recovery before being returned to the dam.

For brain delivery, four‐day‐old hamsters were anesthetized with isoflurane, and then placed onto a brain stereotaxic apparatus (RWD Life science Co., Ltd., Shenzhen, China). During surgery and the virus injection process, hamsters were anesthetized using hypothermia. An incision was made along the midline of the scalp surface, level with the hamster skull along the bregma‐lambda and left–right axes. The coordinates of the site for hippocampus injection in this study were: anteroposterior [A/P] − 1.5 mm; mediolateral [M/L] ± 1.0 mm; dorsoventral [D/V] − 1.5 mm. AAV (1 × 10^12^ v.g./mL, 1.5 μL for each site).

### 2.9 Immunofluorescent staining

Tissues were perfused with 0.9% saline, followed by fixation with 4% paraformaldehyde. Paraffin‐embedded sections (3 μm) were deparaffinized in xylene, rehydrated through graded ethanol to distilled water, and subjected to antigen retrieval in citrate buffer (pH 6.0) at 95℃ for 5 min. After cooling to room temperature, sections were permeabilized with 0.3% Triton X‐100 for 5 min and blocked with normal goat serum for 1 h. The primary antibodies used were anti‐mCherry (Proteintech, Cat# 26765‐1‐AP, Wuhan, China) and anti‐EGFP (Proteintech, Cat# 66002‐1‐Ig, Wuhan, China). Secondary antibodies included Alexa Fluor™ Cyanine5‐conjugated goat anti‐rabbit IgG (Invitrogen, Cat# A10523, Carlsbad, CA, USA) and Alexa Fluor™ 488‐conjugated goat anti‐mouse IgG (Invitrogen, Cat# A‐11001, Carlsbad, CA, USA). Sections were incubated with primary antibodies overnight at 4℃, followed by incubation with secondary antibodies for 1 h at room temperature in the dark. Finally, nuclei were counterstained with DAPI (1:1000) for 15 min.

### 2.10 Identification and analysis of off‐target sites

The potential off‐target sites were analyzed using the online tool (Cas‐OFFinder, http://www.rgenome.net/cas‐offinder/). The mismatch number parameter was set to 3 bp or fewer. To assess potential off‐target activity, a T7EN1 cleavage assay was performed using a previously established protocol.^11^ Briefly, the genomic regions surrounding each candidate off‐target site (OTS) were amplified using primers listed in Table 3. A total of 100 ng purified amplicons were hybridized and digested with T7 Endonuclease I (NEB) in the provided buffer (T7EN1; NEB, Cat. No. M0302L, Ipswich, MA, USA) for 40 minutes. Digested fragments were then visualized via agarose gel electrophoresis, with cleavage products indicating putative mutations. To conclusively validate the possible off‐target issues, the PCR products were cloned and analyzed by Sanger sequencing.

## 3 RESULTS

### 3.1 Generation of *ApoF* conditional alleles using CRISPR/Cas9

Although the CRISPR/Cas9 system has been successfully used to generate gene knockout hamsters via microinjection of one‐cell embryos, precise insertion of DNA fragments at a defined locus remains challenging.^7^, ^11^ In this study, we applied an established two‐cut strategy^11^, ^12^ to create floxed alleles in hamsters using the CRISPR/Cas9 system.

**FIGURE 1:**
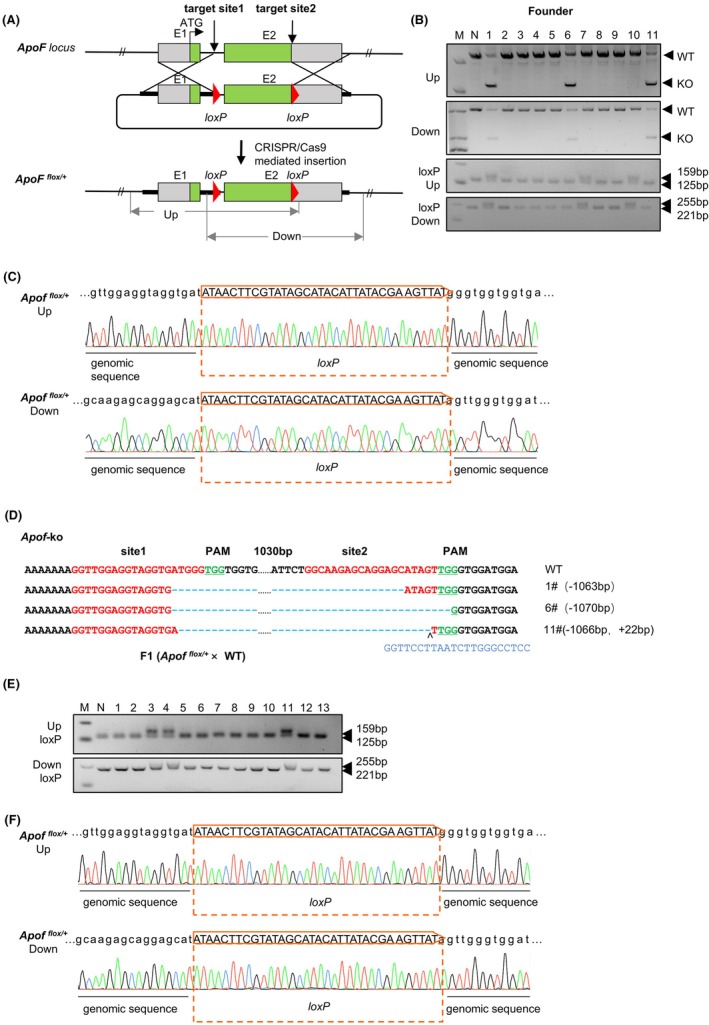
Generation of *ApoF* floxed allele in Syrian hamsters using CRISPR/Cas9. (A) Schematic representation of the targeting strategy. A pair of *loxP* sites was inserted flanking exon 2 to generate the floxed allele. HR‐L and HR‐R denote the left and right homology arms, respectively. (B) PCR genotyping of wild‐type (+/+), heterozygous floxed (flox/+), and homozygous floxed (flox/flox) animals using specific primer sets. (C) Representative sequencing chromatograms confirming the correct insertion of *loxP* sites at both the 5′ and 3′ ends of exon 2. The boxed regions highlight the *loxP* sequences integrated into the genome. (D) Representative sequencing results of CRISPR/Cas9‐edited alleles showing NHEJ‐mediated indels in exon 2 of the target gene. The wild‐type sequence is shown at the top, with the PAM sequences highlighted in green and the gRNA target sites marked in red. Deleted or inserted nucleotides in the mutant alleles are indicated by dashes or additional bases (blue). Founders #1, #6, and #11 exhibited different indel patterns, confirming successful knockout events. (E) PCR analysis demonstrating Cre‐mediated recombination, in which exon 2 flanked by *loxP* sites is excised, yielding the recombined allele. (F) The representative Sanger sequencing results from the F_1_ hamster confirm the correct insertion of loxP sites at both the 5′ and 3′ ends of exon 2. The boxed areas highlight the loxP sequences that have been integrated into the genome.

Apolipoprotein F (ApoF) is a 29‐kilodalton secreted sialoglycoprotein that is a component of HDL and LDL in human serum and plays a role in modulating lipoprotein function.^21^ Detailed gene function of *ApoF* in lipid metabolism is still poorly understood.^22^ To achieve *ApoF* targeting, we designed two sgRNAs to flank exon 2 on both sides (Figure 1A). To facilitate CRISPR/Cas9‐mediated homologous recombination, a donor plasmid template was constructed. It contained exon 2 flanked by two *loxP* sites, with each end bearing a homology arm of approximately 800 bp. Following the transfer of 62 injected zygotes into two pseudopregnant hamsters, 16 pups were born (Table 4). For genotyping the target locus, genetic modifications were assessed using the primer pairs specified in Table 2.

**TABLE 3:** Primers used for detection the potential off‐target sites.

Primer	Sequence (5′‐3′)	Amplicon (bp)
OA1‐S	GACCAAAATGCCTGAGAGAACT	511 bp
OA1‐AS	CTGGCTGAGTGGTGGTCA	
OA2‐S	CAGTAGATCAGTATTCATCCCGAG	557 bp
OA2‐AS	CTTCCATTTGCCTATGATTAAACC	
OA3‐S	GACACATACATGGGACCAAACTAG	539 bp
OA3‐AS	GAGACTTCCCACAGTGAGCAC	
OA4‐S	GCAAGGATGGAGGTGGGTA	467 bp
OA4‐AS	GCATAAGATCCCAGAATACCCA	
OA5‐S	CATCAAGGCTACATCTACTCCAAG	649 bp
OA5‐AS	TACTAGTTACTGTATTGTGGCATGAC	
OA6‐S	GGTAGATCCGAGGCAGTACAAC	564 bp
OA6‐AS	GGTGACCAGAGCAGAGAGGAC	
OB1‐S	GAATCAGTTATCATGGACATTTCTTC	521 bp
OB1‐AS	GTCCAGATCAGAGTCAATATTCG	
OB2‐S	GACACATTCTCTCAGCCTAGATTATC	639 bp
OB2‐AS	CAGGATCAGCAAGACTCAGAGTG	
OB3‐S	CACAGCCAGCCACAGAATAAG	555 bp
OB3‐AS	GATTATCCCTAGGCAAGATGTGATG	
OB4‐S	CATGCAGCAAGGAGAGGTTG	579 bp
OB4‐AS	CTGAAAATGGGAAGTTTGACTG	
OB5‐S	CCCACAAATACCTGTAACTCCAG	700 bp
OB5‐AS	GCAACTTCTACCCTGATTGAGAC	
OB6‐S	CTGCCATCTTACCCGATTACT	600 bp
OB6‐AS	GTGGGTTGACACTCTGGAAC	
OB7‐S	GTGCAATCAAAAGTATGTCCAAC	324 bp
OB7‐AS	CATCAGTGACATGGAGTTCAT	
OB8‐S	GTGAGTAGATGAGAATATTGCTGGT	555 bp
OB8‐AS	CATTCTGAGTGAGTGTGGCC	
OB9‐S	CAGTCTGGTGAGGGATGAGC	484 bp
OB9‐AS	GTGTTTAGTCTGTTGGTGGATTC	
OB10‐S	GTCTCCCCAAAGTGTGTTCCT	465 bp
OB10‐AS	GCACCAAGTCCACTGAACG	
OB11‐S	GTTTAGGGCAGTGGTTCTCAG	515 bp
OB11‐AS	GATCTTGGTTCTACATGATGACAC	
OB12‐S	CATTGAAGTAAGGAGCAGTTCGAG	639 bp
OB12‐AS	GCAGAATCTAGTAACATAGGCATTAAG	
OB13‐S	GCATTCTGAAGAGCTACTTGACC	574 bp
OB13‐AS	GCTACCACTAAAGTCCACCTTG	

**TABLE 2:** Primers used for genotyping in this study.

Primer	Sequence (5′‐3′)	Amplicon (bp)
GH‐ApoF‐F1	GAAATCCAACACCCTCTTGTAGC	2204 bp
GH‐ApoF‐loxP‐R2	GATGACAATAGCCCAAACCCA	
GH‐ApoF‐loxP‐F1	GCCTAGAAATGACGGAGACTAAG	2117 bp
GH‐ApoF‐R1	CAAATTAAATAACAAACAGATACAGACAG	
GH‐ApoF‐loxP‐F1	GCCTAGAAATGACGGAGACTAAG	loxP‐159 bp Wt‐125 bp
GH‐ApoF‐loxP‐R1	GGCCCAAGATTAAGGAACCTC	
GH‐ApoF‐loxP‐F2	CAGCCAGAGATCACGAAGGA	loxP‐255 bp Wt‐221 bp
GH‐ApoF‐loxP‐R2	GATGACAATAGCCCAAACCCA	
GH‐ApoF‐F2	CTGGAGGGTAAGATCAATGCC	Cut‐751 bp Wt‐1848 bp
GH‐ApoF‐R2	CTAATGTCATACCCAAGAATTCAAGC	

To assess genetic modifications, genomic DNA from the offspring was amplified with the primers listed in Table 2. The positions of the PCR primers are shown in Figure S1. Three founder animals (#1, #7, and #10) were found to carry the floxed exon 2 allele, while three others (#1, #6, and #11) contained NHEJ‐induced deletions (Figure 1B). Sanger sequencing validated the precise insertion in founders #1, #7, and #10 (Figure 1C), and confirmed indel mutations in #1, #6, and #11 (Figure 1D).

### 3.2 Germline transmission analysis

To assess germline transmission of *ApoF‐floxed* allele in hamsters, potential founder #10 was mated to a wild‐type hamster and the F_1_ offspring were genotyped. PCR produced target amplicons in the F1 generation that matched the size of those from the founder (Figure 1E). Sanger sequencing further verified that the F1 offspring inherited the same genetic modifications (Figure 1F), demonstrating successful germline transmission of the CRISPR/Cas9‐edited conditional *ApoF* allele.

### 3.3 *Cre/loxP*‐mediated recombination in the heart of *ApoF* ^*flox*^ ^*/+*^ hamsters

**FIGURE 2:**
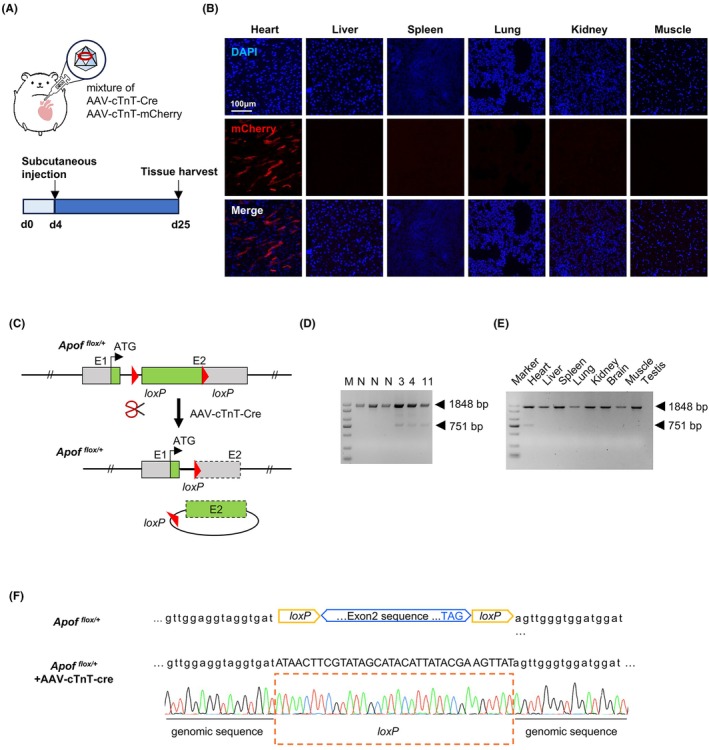
In vivo delivery and validation of the floxed allele with cardiac‐specific Cre recombination. (A) Schematic illustration of subcutaneous AAV injection in hamsters. Diagram of the targeting construct and Cre‐mediated recombination strategy. Exon 2 was flanked by *loxP* sites, allowing excision upon Cre expression. (B) Representative fluorescence imaging of mCherry reporter expression across multiple tissues. Scale bar, 100 μm. Strong mCherry fluorescence was observed exclusively in the heart, while other tissues were negative, confirming cardiac‐specific transgene expression following AAV delivery. (C) Schematic representation of Cre‐mediated excision at *loxP* sites after in vivo delivery of AAV‐Cre. (D) PCR analysis of genomic recombination in the hearts of injected animals and verification of the cardiac specificity of this recombination event. The unedited allele yielded a 1848 bp fragment, whereas the recombined allele produced a 751 bp fragment. (E) PCR analysis across multiple tissues demonstrated that recombination occurred specifically in the myocardium. (F) Sanger sequencing chromatograms verifying the recombined allele and confirming precise Cre‐mediated excision of exon 2.

To test the precise in vivo excision of floxed exon 2 in the *ApoF* locus, *ApoF* ^*flox/+*^ F_1_ hamsters (F_1_ hamster: #3, #4 and #11) received subcutaneous injections of a mixture of AAV‐cTNT‐Cre and AAV‐cTNT‐mCherry (Figure 2A). The mCherry expression confirmed successful and heart‐specific delivery (Figure 2B). A schematic illustration of the expected Cre‐mediated recombination following AAV‐Cre delivery is provided in Figure 2C. Three weeks after AAV administration, the hamster were sacrificed and tissue collected for target recombination validation. PCR analysis of various tissues from treated hamsters showed a truncated DNA fragment exclusively in the heart, indicating successful recombination (Figure 2D,E). Sanger sequencing of these PCR products further verified the precise *Cre/loxP*‐mediated recombination at the *ApoF* gene locus (Figure 2F).

### 3.4 *Cre/loxP*‐mediated recombination in the brain of *ApoF* ^*flox*^ ^*/+*^ hamsters

**FIGURE 3:**
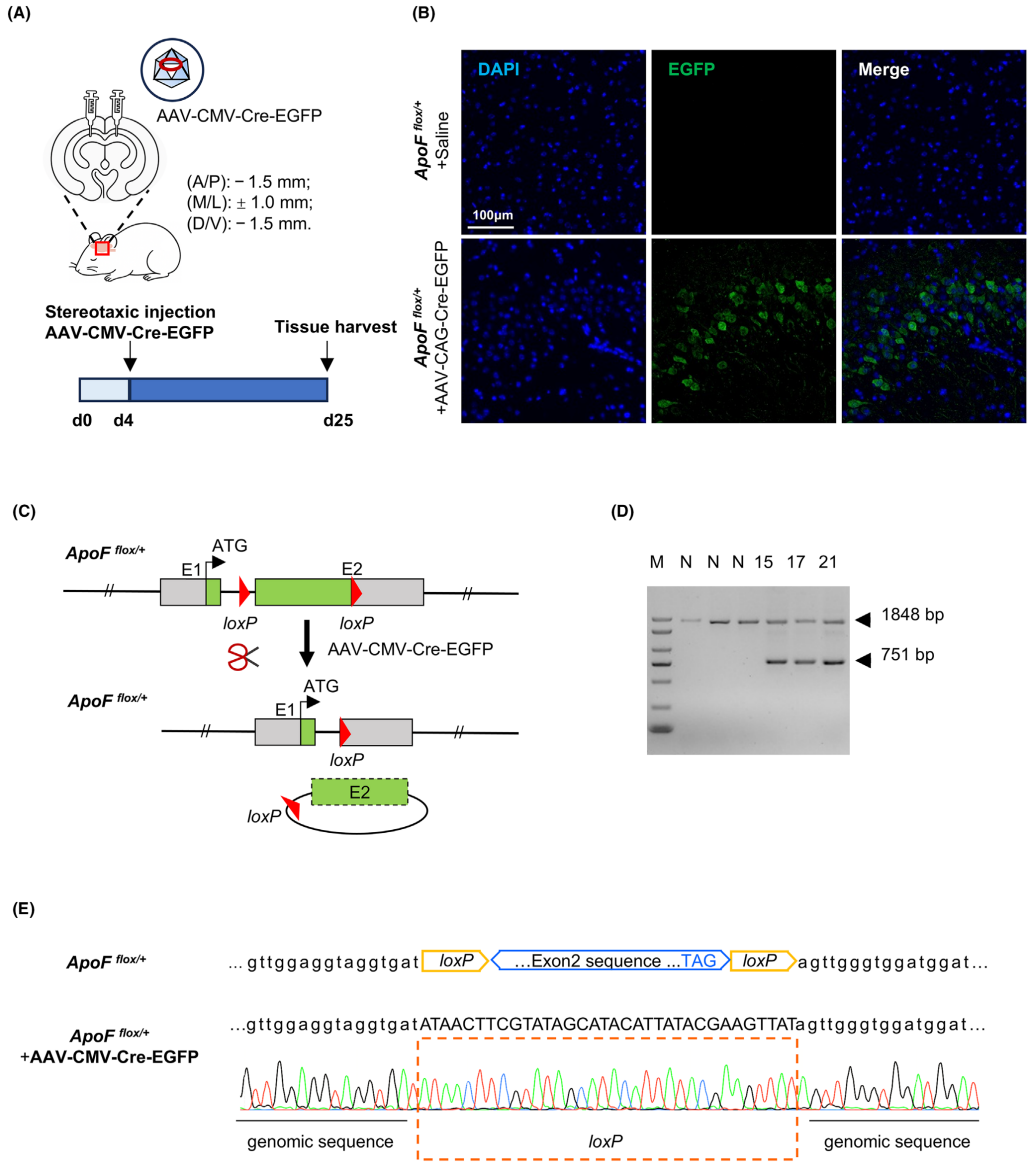
Validation of AAV‐mediated Cre recombination following stereotaxic brain injection. (A) Schematic illustration of stereotaxic AAV injection into the brain of hamsters. (B) Representative fluorescence imaging of the injection site in brain tissue. Scale bar, 100 μm. Strong EGFP fluorescence was observed at the targeted region, indicating robust and localized viral expression following stereotaxic delivery. (C) Schematic representation of Cre‐mediated excision at *loxP* sites after in vivo delivery of AAV‐Cre. (D) PCR analysis of genomic recombination in the brain tissue of injected animals. The unedited allele generated a 1848 bp fragment, whereas the recombined allele yielded a 751 bp fragment. (E) Sanger sequencing chromatogram of the recombined allele, demonstrating precise Cre‐mediated excision of exon 2.

To further validate Cre/*loxP*‐mediated recombination in vivo, AAV‐CMV‐Cre‐EGFP was stereotactically injected into the brain of *ApoF* ^*flox/+*^ hamsters (F_1_ hamster: #15, #17 and #21) (Figure 3A). The EGFP fluorescence was observed at the injection site (Figure 3B), indicating local viral transduction. A schematic representation of Cre‐mediated excision at *loxP* sites following in vivo delivery of AAV‐Cre is shown in Figure 3C. PCR analysis of the targeted tissue amplified the truncated DNA fragment resulting from successful recombination (Figure 3D), which was further confirmed by Sanger sequencing to verify the precise excision at the *ApoF* locus (Figure 3E).

### 3.5 Off‐target analysis

**FIGURE 4:**
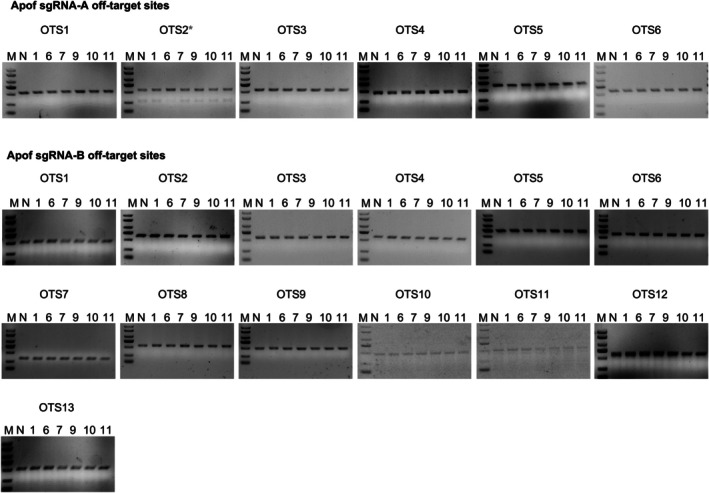
Analysis of the off‐target effects. Potential off‐target mutations were investigated by T7EN1 cleavage assay in #1, #6, #7, #9, #10, and #11F_0_ hamsters. Marker and wild‐type control are located in the left two lanes of the gel. Samples with cleavage bands were marked with asterisks and sub‐cloned for sequencing.

To test the off‐target damage in genetically modified hamsters, a T7EN1 cleavage assay was performed on six F0 animals (#1, #6, #7, #9, #10, #11). The analysis encompassed six predicted off‐target sites for the ApoF‐A sgRNA and thirteen for the ApoF‐B sgRNA. The assay revealed no detectable mutations at any of the 19 examined OTS loci in the selected hamsters, as summarized in Figure 4. The target information and related primers can be found in Table 3.

**TABLE 4:** Summary of the embryo injection and genotyping information.

Target gene	gRNA Con. (ng/μL)	Donor Con. (ng/μL)	Cas9 protein (ng/μL)	No. of injected zygotes	No. of transplanted zygotes	Newborns	No. of pups with Flox	No. of pups with indels
*ApoF*	20	9	20	62	62	11	3	3

## 4 DISCUSSION

The golden Syrian hamster has emerged as a critically important model for human biomedical research. Its physiology, immune responses, and metabolic features more closely resemble those of humans than those of mice, particularly in infectious disease and cardiometabolic studies.^23^, ^24^ However, its utility has been constrained by a limited genetic toolbox. In this study, we successfully established conditional knockout hamsters, moving the model beyond simple constitutive knockouts to sophisticated, spatially and temporally controlled genetic analysis.

The key challenge in hamster genome engineering has been the notoriously low efficiency of HDR‐mediated precise manipulation in zygotes, compounded by the in vitro two‐cell block. While CRISPR/Cas9 facilitated NHEJ‐mediated gene knockouts, the lack of reported HDR‐based knock‐in strategies suggested that this pathway was highly inefficient in hamsters. We employed a preassembled ribonucleoprotein (RNP) complex consisting of Cas9 protein, sgRNAs, and a donor plasmid, rather than the conventional approach of injecting Cas9 mRNA. Most importantly, we implemented a “one‐donor and two‐cut” strategy, which markedly enhanced the efficiency of precise insertion. Our success in achieving precise *loxP* site integration with an efficiency of up to 27% demonstrates that HDR is indeed feasible and can be efficient in hamster zygotes.

The transmission of the floxed alleles through the germline and, most importantly, their successful recombination upon exposure to Cre recombinase in vivo provides definitive functional validation of our model. By using AAV‐mediated Cre delivery (AAV‐cTNT‐Cre for heart and AAV‐CMV‐Cre‐EGFP for brain), we confirmed that the *loxP* sites are correctly positioned and fully functional, excising the target exon and generating the predicted allele deletion in a tissue‐specific manner. This validation step is crucial, as it moves beyond genotypic confirmation to demonstrate phenotypic utility, proving that these hamsters can be used for future conditional gene ablation studies.

Regarding off‐target effects, we analyzed 19 potential off‐target sites (Table 3) and no detectable off‐target activity was identified at the tested loci. Comprehensive off‐target assessment needs further whole‐genome deep sequencing. However, for animal models, off‐target effects are generally not a major concern, as they can be effectively diluted out through breeding strategies.

In summary, we have developed for the first time a CRISPR/Cas9‐based strategy for generating conditional knockout alleles in the golden Syrian hamster. Potential applications of this cKO platform are wide‐ranging. It can be used to interrogate gene function in cardiovascular, metabolic, and infectious disease models for which hamsters are ideal, such as atherosclerosis, diabetes, and respiratory viral infections.^25^ In addition, the system allows rapid testing of therapeutic strategies that require tissue‐specific genetic control, including gene therapy vectors and genome editors. Collectively, these features position the golden Syrian hamster as a versatile bridge between mouse models and higher mammals for translational research. Our work transforms the hamster into a fully genetically tractable organism capable of addressing complex biological questions through precise genetic manipulation. We believe that this achievement will significantly accelerate the use of hamsters in modeling human diseases and testing therapeutic interventions.

## AUTHOR CONTRIBUTIONS

**Wei Chen:** Methodology. **Xu Zhang:** Methodology. **Rui Fan:** Data curation; writing – original draft. **Xia Li:** Methodology. **Feifei Guan:** Methodology. **Gefan Wan:** Methodology; validation. **Weining Kong:** Validation. **Xiaolong Qi:** Methodology; validation. **Shuo Pan:** Methodology. **Sijing Shi:** Methodology. **Yuanlong Su:** Methodology. **Shan Gao:** Methodology. **Wei Huang:** Methodology. **Xunde Xian:** Methodology. **Jiangning Liu:** Conceptualization; supervision. **Yuhui Wang:** Conceptualization; supervision. **Yuanwu Ma:** Conceptualization; supervision.

## FUNDING INFORMATION

This work was supported by CAMS Innovation Fund for Medical Sciences (2022‐I2M‐1‐020, 2021‐I2M‐1‐024 and 2023‐I2M‐2‐001), State Key Laboratory Special Fund (2060204), the Non‐profit Central Research Institute Fund of the Chinese Academy of Medical Sciences (2023‐PT180‐01), Open Research Project in State Key Laboratory of Vascular Homeostasis and Remodeling (Peking University, 202411) and Haihe Laboratory of Cell Ecosystem Innovation Fund (HH24KYZX0007), the National Key Research and Development Program of China from the Ministry of Science and Technology (2021YFF0702802).

## CONFLICT OF INTEREST STATEMENT

Yuanwu Ma and Jiangning Liu are editorial board members of Animal Models and Experimental Medicine (*AMEM*) and authors of this article. To minimize bias, they were excluded from all editorial decision making related to the acceptance of this article for publication.

## ETHICS STATEMENTS

All animal experiments were conducted and approved in accordance with the gudelines of the Animal Care and Use Committees (IACUC) of the Institute of Laboratory Animal Science, Peking Union Medical College (IACUC‐MYW23004).

## Supporting information


**Figure S1.** Positioning diagram of PCR primers. The schematic diagram of primer positions in Table 3.

## Data Availability

All data are available in the main text. The newly generated animal has been deposited in rat resource center (https://ratresource.cnilas.org/) and National Human Diseases Animal Model Resource Center (https://namr.org.cn/). All materials and animal shares are available from the lead corresponding author (mayuanwu@cnilas.org or j2012110009@pumc.edu.cn) upon reasonable request.
