# Supplementary figures and images for: Generating golden Syrian hamsters with conditional alleles via zygote microinjection of CRISPR/Cas9
Source: Animal Model Exp Med. 2025 Nov 14;9(2):308–18. doi: 10.1002/ame2.70107 (PMC13042419; doi:10.1002/ame2.70107)

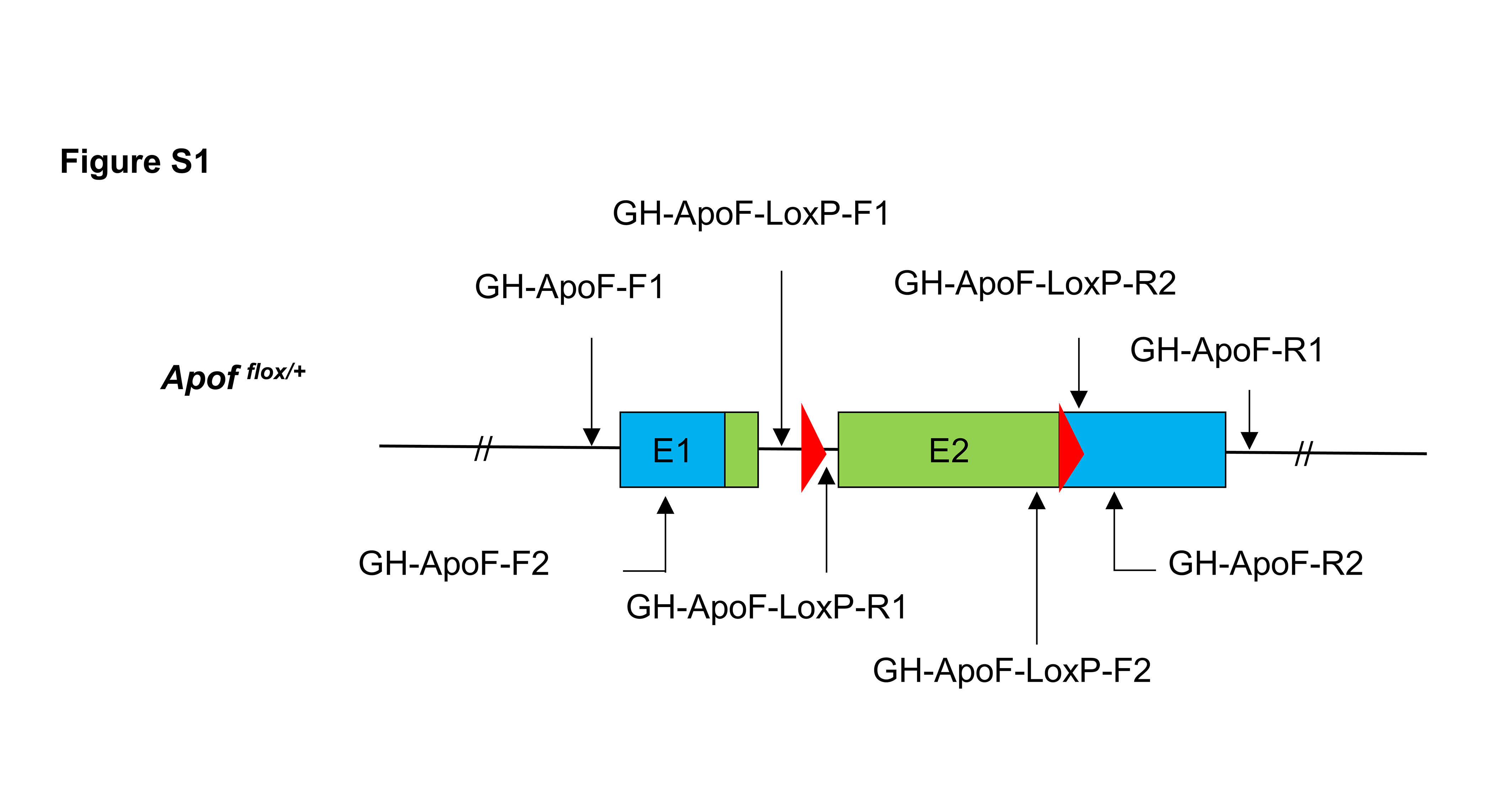

Supplement: Supplementary file 1 — Figure S1. Positioning diagram of PCR primers. The schematic diagram of primer positions in Table 3. [file AME2-9-308-s001.tif]
